# Successful ACL repair by dynamic intraligamentary stabilisation is non‐inferior in functional performance and worse in proprioception compared to healthy controls in a case‐matched study

**DOI:** 10.1002/jeo2.70047

**Published:** 2024-10-26

**Authors:** Sophie A. Gommers, Ajmal Farid, Jeroen de Groot, Inger N. Sierevelt, Daniël Haverkamp, Dutch ACL repair study group

**Affiliations:** ^1^ Department of Orthopaedic Surgery Xpert Clinics Orthopedie Amsterdam The Netherlands; ^2^ Department of Orthopaedic Surgery Bergman Clinics Rijswijk The Netherlands; ^3^ Fysiotherapie Polderkracht Emmeloord The Netherlands; ^4^ Orthopaedic Department Spaarne Gasthuis Academy Hoofddorp The Netherlands

**Keywords:** ACL repair, anterior cruciate ligament, dynamic Intraligamentary stabilisation, functional performance, Hop tests, joint position sense test, ligamys, proprioception

## Abstract

**Purpose:**

The primary aim of this study was to assess non‐inferiority in functional performance of the knee after dynamic intraligamentary stabilisation (DIS) surgery at a minimal follow‐up of 1 year compared to healthy controls, based on limb symmetry index (LSI) of the single leg hop test (SLH). Additionally, functional performance based on the single leg triple hop test (SLTH) and side hop test (SH), proprioception and subjective outcome were evaluated.

**Methods:**

A total of 45 DIS patients were 1‐to‐1 matched to a healthy control. Functional performance was evaluated by LSI and absolute values on the SLH, SLTH and SH. Proprioception was assessed by joint position sense (JPS) test and International Knee Documentation Committee (IKDC) scores were obtained.

**Results:**

Non‐inferiority in functional performance after DIS compared to healthy controls was confirmed based on the mean LSI of the SLH and SLTH (97.6% vs. 99.6% and 97.5% vs. 100.6%, respectively) and non‐confirmed on the SH (98.8% vs. 100.0%, respectively). No significant differences were found in absolute value of the SLH and SLTH and a significantly higher absolute value of the SH was found in the DIS group (*p* = 0.01). JPS absolute angular error was significantly higher in the DIS group compared to the control group (*p* = 0.01). The median IKDC score of the DIS group was significantly lower (92, IQR 85–95) than the control group (100, IQR 99–100), *p* < 0.001.

**Conclusions:**

In conclusion, functional performance after DIS was confirmed non‐inferior compared to healthy controls based on the SLH and SLTH, although non‐confirmed on the SH.

**Level of evidence:**

Level III

**Abbreviations**
AAE: absolute angular error
ACL: anterior cruciate ligament
ACLR: anterior cruciate ligament reconstruction
BMI: body mass index
CI: confidence interval
cm: centimetre
DIS: dynamic intraligamentary stabilisation
ICC: intraclass correlation coefficient
IQR: interquartile range
JPS: joint position sense test
LSI: limb symmetry index
n: number
SD: standard deviation
SH: side hop test
SLH: single leg hop test
SLTH: single leg triple hop test

## INTRODUCTION

Anterior cruciate ligament (ACL) repair by dynamic intraligamentary stabilisation (DIS) applies dynamic augmentation and stabilisation after ACL rupture. The technique creates an opportunity for the ruptured ACL to heal and allows full range of motion and full weightbearing directly after surgery [25]. Varying failure rates of 4%–30% up to 5 years follow‐up are reported, as well as comparable outcomes to ACLR on anteroposterior laxity, subjective outcome and Tegner score [2, 10, 33].

Functional performance after ACL injury and/or surgery is evaluated by hop tests and is scored as limb symmetry index (LSI) by comparing the operated leg to the contralateral leg and an LSI > 90% is recommended as cut‐off for safe return to sport. An LSI of 99% is reported in healthy subjects, which is 9% above this cut‐off [1, 31]. However, evaluation of LSI is under debate as decreased hop performance of the contralateral leg is reported [53]. Therefore, absolute value and number of hops should be compared to healthy controls as well [17, 41], which is lacking in the current literature on DIS. Additionally, a correlation between proprioception and hop test performance is described in the literature and proprioception is commonly evaluated by the joint position sense test (JPS) [16, 49]. Although the idea of successful ACL‐repair is preservation of the native ACL with its mechanoreceptors and therefore leading to restoration of proprioception, there is currently no literature evaluating proprioception after DIS.

To investigate if functional outcome and proprioception are restored after successful ACL‐repair by DIS, comparison with a healthy leg is needed. Since comparison with the contralateral leg is under debate, comparison with healthy subjects is required. Therefore, the primary aim of this study was to assess non‐inferiority in functional performance (LSI of the SLH) of the knee after successful ACL‐repair by DIS at a minimal follow‐up of 1 year compared to case‐matched healthy controls. Secondary, the groups were compared on functional performance using other hop tests (SLTH and SH) and on proprioception (JPS). Evaluation of the subjective outcome was assessed by International Knee Documentation Committee (IKDC) score. We hypothesised the DIS‐group would be non‐inferior in functional performance compared to case‐matched healthy controls and show comparable proprioception.

## METHOD

### Study design

The study was designed as a non‐inferiority case‐matched study in subjects who have been operated with DIS, compared with a healthy case‐matched control group. Comparison with a case‐matched control group was required as decreased hop performance of the contralateral leg is reported and therefore comparison of the operated leg versus contralateral leg is under debate [17, 53]. The study was approved by the Medical Ethical Committee of the Amsterdam medical Centre (NL64718.048.18). and registered at the Nederlands Trial Register (NTR) (trial number NTR7486).

### Study population

**Figure 1:**
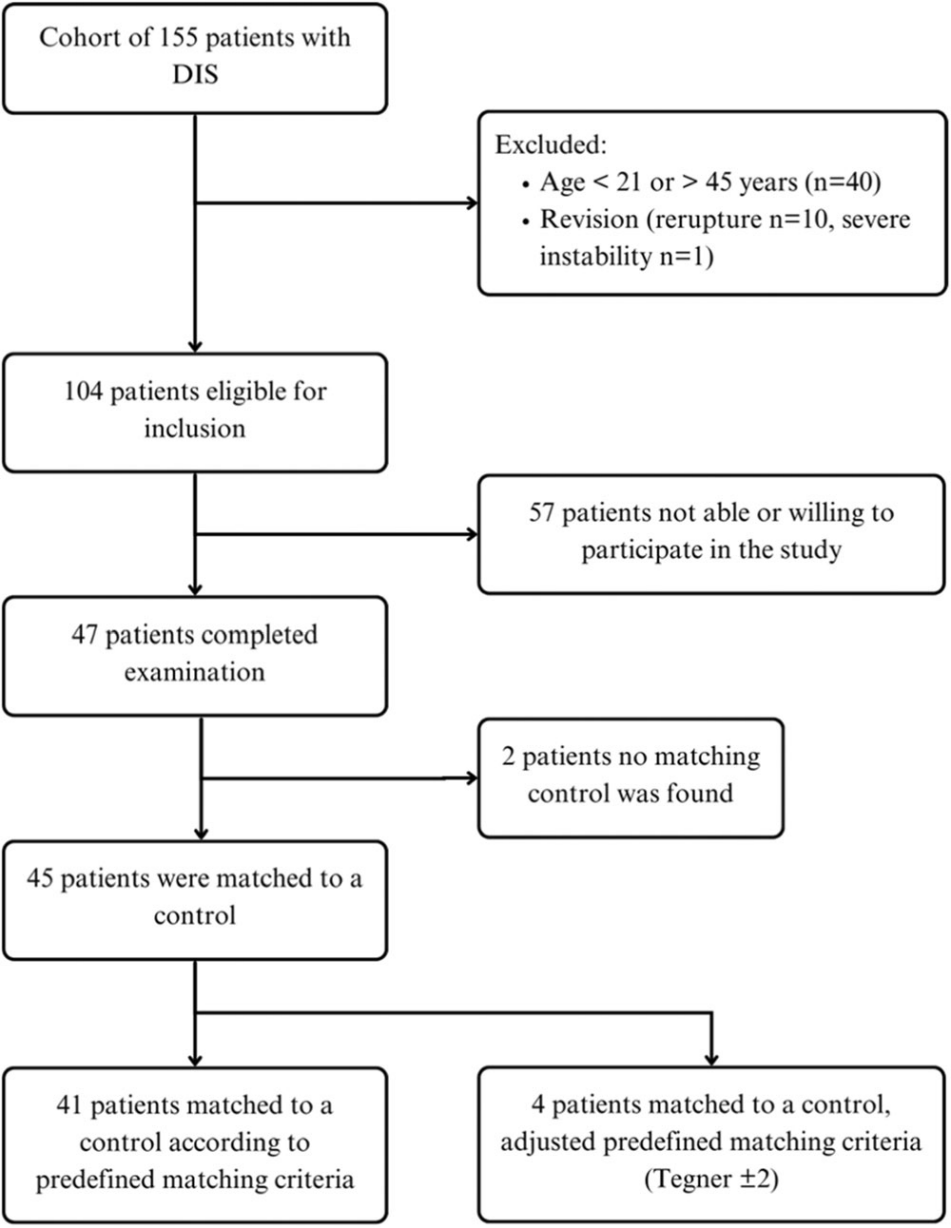
Flow chart of participants assessed for eligibility, examination and matching. DIS, dynamic intraligamentary stabilisation.

The participant flow is shown in Figure 1. All DIS patients were from a prospective case study on failure rate of DIS in 155 consecutive patients with ACL‐rupture (NTR***) [*]. Inclusion criteria were an acute primary rupture of the ACL confirmed by MRI and surgical intervention planned within 21 days, age between 18 and 50 years, and a BMI < 35. Exclusion criteria were osteoarthritis Kellgren–Lawrence grade ≥ 2, traumatic cartilage lesion requiring cartilage repair procedure or degenerative cartilage lesions (Outerbridge grade > 2 and defect size > 1cm^2^), combined ligament injury, pregnancy, rheumatoid arthritis, instability of the contralateral leg, or unwillingness to follow the rehabilitation programme. For the present study, patients were excluded in case of rerupture, revision of DIS or severe instability [20, 45]. For each included patient a matched control was searched according to predefined matching criteria: age (±5 years), gender, body mass index (BMI) (±3 kg/m^2^), type of sports (pivoting/non‐pivoting) and Tegner level (±1). Controls were recruited through connections at local sports clubs and screened on predefined matching criteria.

### Surgical technique and rehabilitation

DIS procedures were performed by 10 experienced surgeons in five participating centres arthroscopically using a previously described surgical technique [7]. The ruptured ACL was sutured and stabilised by use of a polyethylene braided cord that was anchored on the antero‐medial aspect of the tibia by a Monoblock, a spring‐screw implant (Ligamys; Mathys Ltd.), and pre‐loaded with 60–80 N [46]. In case of concomitant meniscus injuries, partial debridement or repair was performed[*].

Patients followed postoperative rehabilitation according to physiotherapy rehabilitation guidelines. During the first week full weight bearing was allowed using a brace in patients without concomitant injury. At three weeks strength training and at ten weeks running and sport specific training exercises were allowed[*].

### Procedures

After confirmed eligibility and signed informed consent, patients and controls were invited to a location of preference (clinic or sport accommodation) for examination, carried out by two trained observers. Patients and controls were manually checked on knee stability by KT1000. Side‐to‐side difference (∆ATT) was calculated as follow: ∆ATT = operated leg‐contralateral leg. The ∆ATT of the matched controls was calculated considering the dominance of the affected knee of the matched case. Examination started with a warming up of 20 squats and 5 squat jumps and between the tests there was a 2‐min rest. The European Board of Sports Rehabilitation recommends a combination of two maximal and one endurance hop tests [50]. The examination procedure consisted of SLH and SLTH as maximal tests, and SH as endurance test [19, 44]. SLH is the most frequent reported hop test in the literature and performance in the SLTH is identified as a predictor of second ACL injury [1, 38]. SH is required to reveal deficits of the operated leg in a fatigued state [14, 39, 40]. The hop tests were followed by a JPS. To measure knee flexion during the JPS, the participant got non‐reflective markers on the greater trochanter, lateral epicondyle of the femur and lateral malleolus, and the tests were videorecorded. Additionally, an IKDC questionnaire was filled out.

### Primary outcome measure; LSI of the SLH

**Figure 2:**
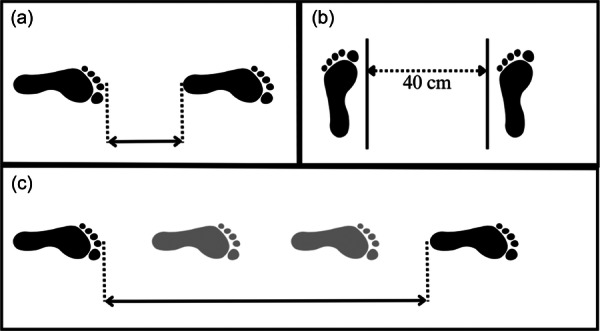
Hop tests. (a) Single leg hop test (SLH), (b) side hop test (SH) and (c) single leg triple hop test (SLTH).

Subjects were asked to jump as far as possible on one leg from a predetermined line and to land on the same leg with hands placed on the back. The distance was measured in centimetres from toe at the push‐off to the heel where the subject landed in one decimal accurate (Figure 2a). The test was performed twice on each leg and the best result counted. The LSI for the SLH was calculated as follow: LSI = (test score affected leg/test score non affected leg) × 100%. As limb dominance influences the limb symmetry, the LSI of the matched controls was calculated considering the dominance of the affected knee of the matched case.

### Secondary outcome measures

**Figure 3:**
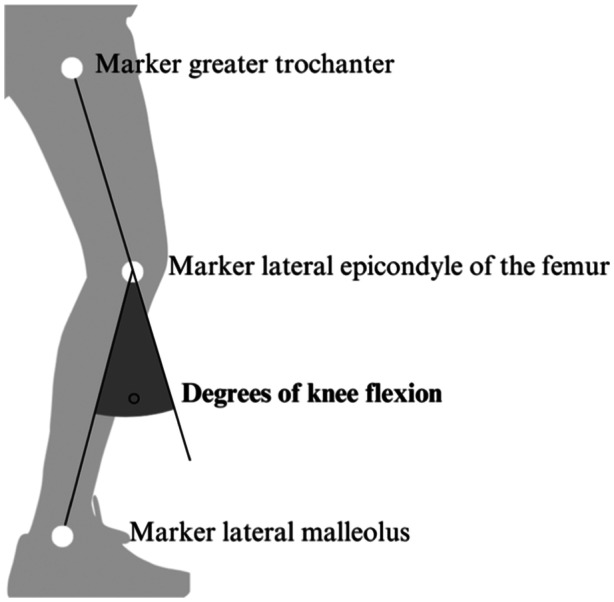
Measurement of knee flexion during the joint positions sense test.

a. *SLH; hop distance*
  For DIS‐patients the best result of the hop distance in centimetres of the operated and contralateral leg were reported. For controls, the best result of hop distance of the (non)dominant leg was used, based on the dominance of the affected knee of the matched case.
b. *SLTH; LSI and hop distance*
  Subjects were asked to jump as far as possible on one leg with the help of three powerful jumps on the same leg with hands place on the back, from a determined line. The distance was measured in centimetres from toe at the push‐off to the heel where the subject landed in one decimal accurate (Figure 2c). The test was performed twice on each leg and the best result counted. The calculated LSI and absolute value of jump distance were reported. For controls, the best result of hop distance of the (non)dominant leg was used, based on the dominance of the affected knee of the matched case.
c. *SH; LSI and number of hops*
  Subjects were asked to make as many side jumps as possible over a period of 30 seconds over two lines that were 40 cm apart, each starting on the same side (Figure 2b). The correct number of jumps without hitting the tape was noted. The test was performed twice on each leg and the best result counted. The calculated LSI and the absolute number of hops was used. For controls, the best result of number of hops of the (non)dominant leg was used, based on the dominance of the affected knee of the matched case.

d. *JPS; absolute angular error*
  Subjects stood blindfolded while holding on to a sturdy chair and were asked to drop actively through the knee. When the examinator measured 50° of knee flexion, the subjects were asked to hold and try to remember the position. After 5 seconds the subjects were asked to return to starting position. Next, the subjects were asked to return as accurately as possible to the remembered position, hold it for 3 s and return to starting position. The test was repeated three times with a pause of 2 s between the repetitions. Proprioception was evaluated by use of the absolute angular error (AAE) of the JPS. First, the reached knee flexion angles (Figure 3) during the JPS were calculated by use of Kinovea® (version 0.8.15, intraclass correlation coefficient > 0.85) [11]. Then, the AAE of the JPS was calculated as follow: AAE = [(target position – trial 1) + (target position – trial 2) + (target position – trial 3)]/3.

e. *Subjective outcome; IKDC*
  Subjects were asked to fill out the IKDC form.

### Sample size calculation

Sample size calculation for non‐inferiority compared to the case matched controls was based on expected mean LSI of 99% in healthy subjects and 92% in patients after ACL repair [1, 31]. Since a cut‐off value of 90% of the LSI for hop performance is recommended by the European Board of Sports Rehabilitation to decrease the risk of subsequent ACL injury after returning to sport, a non‐inferiority limit of 9% is chosen not to exceed this value [18, 28, 50]. Therefore, based on an expected difference of 7% (99%‐92%), a non‐inferiority limit of 9%, a standard deviation (SD) of 3%, a power of 90% and a one‐sided significance level of 0.025, a sample of 48 patients in each group was required to assess non‐inferiority on the SLH [31]. Non‐inferiority limit of the SLTH and SH was based on the pre‐defined non‐inferiority limit of the SLH.

### Statistical analysis

Statistical analysis was performed by use of IBM SPSS statistics 26.0 (IBM Corp.). Demographic and clinical characteristics are described according to their distribution. Continuous data is presented as means with SD in case of normal distribution, otherwise medians with interquartile ranges (IQR) are presented. Categorial data is presented as numbers with accompanying proportions. Comparison of all test variables of the DIS group with the control group was performed by use mixed model analysis to account for the correlated structure of the matched pairs. Crude as well as adjusted differences were calculated and presented with 95% confidence intervals (CI). Adjustment was performed for potential confounders such as age, BMI and Tegner score. DIS was considered non‐inferior to healthy controls, based on the LSI of the SLH, if the lower boundary of the two‐sided 95% confidence interval of the difference between the groups laid beneath the predefined non‐inferiority margin of −9%. Due to skewed distribution of the IKDC score, difference between the two groups was tested by use of the Wilcoxons Signed Ranks test. Differences between operated and contralateral leg in mean scores of hop tests and AAE of JPS were assessed by use of paired t‐tests. Intra‐observer reliability of the JPS measurements was determined by calculation of the intraclass correlation coefficient (ICC, two‐way random effect model, absolute agreement) using the data of the three separate tests. Proportion of patients achieving at least the 90% LSI limit was compared by use of chi^2^ tests. A *p*‐value < 0.05 was considered statistically significant.

## RESULTS

### Subject characteristics

Subject characteristics are presented in Table 1. Due to limited availability of included patients and 1‐to‐1‐matched controls, 45 patients and controls were included in the study. The groups were comparable according to the values of the matching variables and their variability. A significantly higher side‐to‐side difference measured by KT1000 was found in the DIS group (1.9 ± 2.1) compared to the control group (0.7 ± 2.4), *p* = 0.04.

**Table 1:** Subject characteristics.

	DIS group (*n* = 45)	Control group (*n* = 45)	*p*‐value
Age (year), mean ± SD	32.5 ± 9.3	30.4 ± 8.9	0.26
Male, *n* (%)	26 (58%)	26 (58%)	‐
BMI (kg/m^2^), mean ± SD	24.8 ± 3.7	24.5 ± 3.5	0.68
Tegner, mean ± SD	6.2 ± 2.0	6.1 ± 1.6	0.86
Pivoting sports, *n* (%)	19 (42%)	19 (42%)	‐
Follow‐up (months), median (IQR)	31 (25–37)	‐	‐
KT1000 (∆ATT), mean ± SD	1.9 ± 2.1	0.7 ± 2.4	0.04*
*Tear location*			
Proximal, *n* (%)	26 (57.8%)	‐	‐
Mid substance, *n* (%)	13 (28.9%)	‐	‐
Unknown, *n* (%)	6 (13.3%)	‐	‐

Abbreviations: ∆ATT, side‐to‐side difference; IQR, interquartile range; *n*, number; SD, standard deviation.

* Statistically significant.

### Outcome measures

#### Primary outcome measure; LSI of the SLH

The mean LSI of the SLH of the DIS and control group was 97.6% and 99.6%, respectively. With an adjusted difference of −1.52 (95%CI: −8.14; 5.10), non‐inferiority was confirmed (Table 2).

**Table 2:** Outcome measures of hop tests and joint position sense test of DIS and control group.

	DIS group (mean ± SD)	Control group (mean ± SD)	Crude *β* (95%CI)	*p*‐value	Adjusted *β* (95%CI)	*p*‐value
*Hop tests, LSI*						
SLH (%)	97.6 ± 18.3	99.6 ± 12.4	−2.09 (−8.65; 4.46)	0.53	−1.52 (−8.14; 5.10)	0.65
SLTH (%)	97.5 ± 10.5	100.6 ± 10.0	−3.14 (−7.44; 1.16)	0.15	−3.19 (−7.55; 1.16)	0.15
SH (%)	98.8 ± 16.0	100.0 ± 21.6	−1.18 (−9.17; 6.80)	0.77	−1.17 (−9.36; 7.03)	0.78
*Hop tests, affected leg*						
SLH (cm)	93.4 ± 30.6	100.0 ± 28.5	−6.5 (−18.9; 5.9)	0.30	−3.6 (−14.7; 7.4)	0.51
SLTH (cm)	345.9 ± 85.1	360.1 ± 85.7	−14.2 (−50.0; 21.5)	0.43	−8.1 (−39.3; 23.0)	0.61
SH (n)	41.2 ± 14.9	35.7 ± 13.5	5.5 (−0.42; 11.5)	0.07*	6.5 (1.3; 11.6)	0.01*
*Joint position sense test, affected leg*						
AAE (degrees)	6.9 ± 5.0	4.1 ± 3.6	2.8 (0.95; 4.6)	0.003*	2.7 (0.9; 4.6)	0.01*

*Note*: The LSI of the matched controls was calculated considering the dominance of the affected knee of the matched case.

Abbreviations: AAE, absolute angular error; CI, confidence interval; cm, centimetre; LSI, limb symmetry index; *n*, number; SD, standard deviation; SH, side hop test; SLH, single leg hop test; SLTH, single leg triple hop test.

* Statistically significant.

#### Secondary outcome measures

a. *LSI of the SLTH and SH*
  The mean LSI scores are reported in Table 2. The mean LSI of the SLTH of the DIS and control group was 97.5% and 100.6%, respectively. With an adjusted difference of −3.19 (95%CI: −7.55; 1.16), non‐inferiority confirmed. The mean LSI of the SH of the DIS and control group was 98.8% and 100.0%, respectively. With an adjusted difference of −1.17 (95%CI: −9.36; 7.03), non‐inferiority was non‐confirmed. In the DIS group 57.8% of the patients scored LSI > 90% on the SLH, SLTH and SH, and in the control group 53.3%, which was not significantly different (Table 3).
b. *Mean scores of the SLH, SLTH and SH*
  The mean scores of the hop tests of the DIS and control group are reported in Table 2, scores of the operated and contralateral leg are reported in Table 4. No significant differences were found in mean distance of the SLH and the SLTH of the DIS versus control group and the operated versus contralateral leg. The mean number of hops of the SH in the DIS group was significantly higher than the control group with an adjusted difference of 6.5 (95%CI: 1.3; 11.6). No significant difference was found in mean number of hops of the SH between the operated and contralateral leg.
c. *Proprioception: JPS*
  The DIS group had a significantly higher AAE than the control group, with an adjusted difference of 2.7 (95%CI: 0.9; 4.6) (Table 2). No significant difference was found in AAE between the operated and contralateral leg (Table 4). An ICC of 0.81–0.94 was found.
d. *Subjective outcome: IKDC*
  The median IKDC score of the DIS and control group was 92 (IQR 85–95) and 100 (IQR 99–100), respectively. The difference was statistically significant (*p* < 0.001).

**Table 3:** Cases with LSI scores > 90% on hop tests.

	DIS group (*n* (%))	Control group (*n* (%))	Pearson Chi‐Square	*p*‐value
SLH	33 (73.3%)	35 (77.8%)	0.241	0.62
SLTH	38 (84.4%)	39 (86.7%)	0.090	0.76
SH	35 (77.8%)	31 (86.9%)	0.909	0.34
SLH & SLTH & SH	26 (57.8%)	24 (53.3%)	0.180	0.67

Abbreviations: LSI, limb symmetry index; *n*, number; SH, side hop test; SLH, single leg hop test; SLTH, single leg triple hop test.

**Table 4:** Outcome measures of hop tests and joint position sense test of operated and contralateral leg.

	DIS operated leg	DIS contralateral leg		
	(mean ± SD)	(mean ± SD)	Difference (95% CI)	*p*‐value
*Hop tests*				
SLH (cm)	93.4 ± 30.6	96.8 ± 20.8	−3.4 (−8.2; 1.5)	0.17
SLTH (cm)	345.9 ± 85.1	354.0 ± 75.2	−8.2 (−19.7; 3.4)	0.16
SH (*n*)	41.2 ± 14.9	42.2 ± 15.2	−0.93 (−2.8; 0.9)	0.31
*Joint position sense test*				
AAE (degrees)	6.9 ± 5.0	5.6 ± 5.0	1.3 (−0.4; 3.0)	0.14

Abbreviations: AAE, absolute angular error; CI, confidence interval; cm, centimetre; LSI, limb symmetry index; *n*, number; SD, standard deviation; SH, side hop test; SLH, single leg hop test; SLTH, single leg triple hop test.

## DISCUSSION

The present study found non‐inferiority in functional performance after successful DIS surgery compared to matched healthy controls at a median follow‐up of 31 months (IQR 25–37), based on the LSI of the SLH. This confirms our hypothesis. Non‐inferiority was also confirmed based on the LSI of SLTH, however not for the LSI of the SH. Functional performance on the SLH and SLTH was comparable between both groups, with an even better score on the side hop test in the DIS group. Absolute hop test scores of the operated leg were overall comparable to the contralateral leg. Worse proprioception measured with the JPS and IKDC‐score were found in the DIS group compared to the control group.

Found mean LSI scores are in line with previous literature on DIS [7, 22]. In accordance with previous literature on ACLR, on all hop tests combined only 58% of the DIS‐patients and 53% of the controls had an LSI above 90% [54]. This questions the use of this criteria for safe return to sport, as it suggests that hop symmetry may not indicate ideal biomechanics. Besides that, LSI for functional performance is under debate as decreased hop performance of the contralateral leg is reported, leading to falsely high LSI [17, 29, 53, 54]. However, the use of hop distance to assess functional performance of the knee after ACL‐injury for return to sport testing is also questioned, because it is primarily a function of the hip and ankle, with a reported contribution of the knee of 12%. Interestingly, the reported contribution of the knee during the landing phase of the SLH is 65% [26]. Altered biomechanics are seen in the injured and contralateral leg of ACLR‐patients, causing larger stress on the ACL, possibly causing a higher risk on (re‐)rupture [27, 30, 42]. Biomechanical analysis might be helpful during rehabilitation, although it requires specialised equipment and analysis, and strong evidence on associations between altered biomechanics and clinical outcomes is lacking. Meanwhile, symmetry on hop tests and therefore findings of the present study should be interpreted with cause as it might cover for abnormal knee kinetics and kinematics that might lead to an increased risk of re‐rupture or contralateral ACL‐injury.

Although the native ACL with its mechanoreceptors was preserved by DIS, proprioception was worse than the control group. Interestingly, the operated leg was comparable to the contralateral leg. This might suggest that worse proprioception existed prior to injury and/or DIS‐surgery affects the proprioceptive capacity of the injured and non‐injured leg [3]. However, the reported difference of 2.7° between DIS patients and controls should be interpreted with cause since clinical significance is uncertain [16, 43]. Previous studies using a similar weightbearing JPS found that proprioception in a non‐fatigued state after ACLR was similar to controls at a lower target angle of 15° and 30°, although a worse proprioception was found at a higher target angle of 60° [8, 34]. Even more, the present study tested DIS‐patients and controls in a fatigued state, conversely to ACLR‐patients in previous literature, which influences knee proprioception [35]. Therefore, future research on proprioception after DIS versus ACLR is needed to deduce a sound conclusion on the theoretically improvement of proprioception after DIS, preferably using a similar test protocol using several target angles.

The found IKDC score of the DIS group was consistent with those reported in the literature for DIS at 2 and 5 years postoperative [15, 22, 23, 24, 47]. The reported difference between DIS‐patients and healthy controls might be clinically important, based on MCID scores of 7.1–13.9 reported in the literature [4, 5, 36, 37, 51]. However, the mean score after DIS is above the patient acceptable symptom state threshold of 73.6–88.6, reported 2 years after ACL‐repair [4, 12]. Even more, a recent meta‐analysis showed no significant difference on IKDC between DIS and ACLR [33]. To conclude, the IKDC score after DIS might be worse compared to healthy controls, yet it seems acceptable for surgical treatment of ACL‐injury.

Although long‐term outcomes of DIS compared to ACLR are lacking, a recent meta‐analysis showed comparable subjective outcomes, failure rates and re‐rupture rates based on studies with a follow‐up of 1–5 years [33]. Even more, comparable results are reported based on LSI on hop tests, LSI on quadriceps and hamstrings force and biomechanical pattern during gate [22, 47]. Advantages of DIS compared to ACLR are no donor‐site morbidity and theoretically restored proprioception. The latter is questioned by results of the present study as proprioception was worse than healthy controls and research comparing proprioception between DIS and ACLR is needed. As DIS‐surgery is performed within 21 days after injury, advantages compared to ACLR are earlier return to work of nearly one‐month, shorter duration of physical therapy and immediate meniscal repair if necessary, which might lead to a lower rate of osteoarthritis [6, 48, 52]. However, an important disadvantage of the short time between injury and surgery is that it eliminates the possibility to start with conservative treatment first, which has been reported to avoid surgery in 61% of the patients [13]. Therefore, strict patient selection for DIS is crucial to avoid unnecessary surgery. It is already reported that higher failure rates after DIS are associated with non‐proximal tears, young age and participation in high level sports [2, 15, 21]. Revision to ACLR is possible in case of failure, although two‐stage ACLR revision is reported in the literature due to arthrofibrosis and excessive tibial tunnel enlargement following the removal of the monoblock [9]. DIS needs to prove itself in future long‐term follow‐up studies on failure, contralateral ACL‐rupture, osteoarthritis, functional performance and subjective outcome to become a good alternative to ACLR.

This study has several limitations. First, a sample of 48 patients in each group was required to assess non‐inferiority, only 45 patients were included in each group including four matches with a Tegner ±2 instead of ±1. Although non‐inferiority was confirmed based on the LSI of the single leg hop test and single leg triple hop test, non‐inferiority was non‐confirmed based on the LSI of the side hop test, which could hypothetically be confirmed with inclusion of 48 patients. Second, additional matching criteria as sports level and sport intensity (hours per week) might have enhanced the methodological strength, however due to matching on (non‐)pivoting sport and Tegner we expect the risk on selection bias to be minimised. Third, heterogeneity of the DIS group might lead to variability of test results, however the impact on the reported difference between patients and controls was minimised due to the 1‐to‐1 matching. Fourth, the examinator was not blinded, which might have introduced information bias. Fifth, DIS patients went to a physiotherapist by preference, which may have led to different rehabilitation strategies and thereby differences in functional outcome and proprioception [32].

This study's strength is that it is a multicenter study with matched controls to account for possible confounders. Additionally, it is the first study to compare functional outcome after DIS with healthy controls, and to evaluate proprioception after DIS. Even more, functional outcome was not only compared by LSI, but also by absolute values of hop distance and number of hops.

## CONCLUSION

Functional performance after DIS was confirmed non‐inferior compared to case‐matched healthy controls based on the SLH and SLTH and non‐confirmed on the SH and absolute scores on hop tests were equal or even better in the DIS group. However, proprioception was worse compared to case‐matched healthy controls despite preservation of the mechanoreceptors, although interpretation with cause is needed since clinical significance is uncertain.

## DUTCH ACL REPAIR STUDY GROUP

Niels Baas, Bergman Clinics. Maaike v/d Borne, Amphia Ziekenhuis. Hans Frejlach, Acibadem Medical Centre. Peter Joosten, Amphia Ziekenhuis. Tom Hogervorst, Bergman Clinics Orthopedie. Daniël Hoornenborg, Xpert Clinics Orthopedie. Gino Kerkhoffs, Amsterdam UMC. Arno van Lieshout, Bergman Clinics. Bart Muller, Xpert Clinics Orthopedie. Marina van Rhee, Xpert Clinics Orthopedie. Harm van der Vis, Xpert Clinics Orthopedie.

## AUTHOR CONTRIBUTIONS

All authors contributed substantially.

## CONFLICT OF INTEREST STATEMENT

The authors declare no conflict of interest.

## ETHICS STATEMENT

Informed consent was obtained from all individual participants included in the study. This study was performed in line with the principles of the Declaration of Helsinki. Approval was granted by the Ethics Committee of the Amsterdam Medical Centre (No. NL64718.048.18).

## Data Availability

The data that support the findings of this study are available from the corresponding author upon reasonable request.

## References

[1] Abrams, G.D. , Harris, J.D. , Gupta, A.K. , McCormick, F.M. , Bush‐Joseph, C.A. & Verma, N.N. (2014) Functional performance testing after anterior cruciate ligament reconstruction: a systematic review. Orthopaedic Journal of Sports Medicine, 21(1), 2325967113518305. Available from: 10.1177/2325967113518305/FORMAT/EPUB PMC4555525 26535266

[2] Ahmad, S.S. , Schreiner, A.J. , Hirschmann, M.T. , Schröter, S. , Döbele, S. , Ahrend, M.D. et al. (2019) Dynamic intraligamentary stabilization for ACL repair: a systematic review. Knee Surgery, Sports Traumatology, Arthroscopy, 27(1), 13–20. Available from: 10.1007/s00167-018-5301-z 30474692

[3] Arockiaraj, J. , Korula, R.J. , Oommen, A.T. , Devasahayam, S. , Wankhar, S. , Velkumar, S. et al. (2013) Proprioceptive changes in the contralateral knee joint following anterior cruciate injury. The Bone & Joint Journal, 95–B(2), 188–191. Available from: 10.1302/0301-620X.95B2.30566 23365027

[4] Batista, J.P. , Maestu, R. , Barbier, J. , Chahla, J. & Kunze, K.N. (2023) Propensity for clinically meaningful improvement and surgical failure after anterior cruciate ligament repair. Orthopaedic Journal of Sports Medicine, 11, 232596712211468. Available from: 10.1177/23259671221146815 PMC10102942 37065184

[5] Beletsky, A. , Naami, E. , Lu, Y. , Polce, E.M. , Chahla, J. , Okoroha, K.R. et al. (2021) The minimally clinically important difference and substantial clinical benefit in anterior cruciate ligament reconstruction: a time‐to‐achievement analysis. Orthopedics, 44(5), 299–305. Available from: 10.3928/01477447-20210819-03 34590953

[6] Bieri, K.S. , Scholz, S.M. , Kohl, S. , Aghayev, E. & Staub, L.P. (2017) Dynamic intraligamentary stabilization versus conventional ACL reconstruction: a matched study on return to work. Injury, 48(6), 1243–1248. Available from: 10.1016/j.injury.2017.03.004 28318538

[7] Büchler, L. , Regli, D. , Evangelopoulos, D.S. , Bieri, K. , Ahmad, S.S. , Krismer, A. et al. (2016) Functional recovery following primary ACL repair with dynamic intraligamentary stabilization. The Knee, 23(3), 549–553. Available from: 10.1016/j.knee.2016.01.012 26972809

[8] Büyükafşar, E. , Başar, S. & Kanatli, U. (2020) Proprioception following the anterior cruciate ligament reconstruction with tibialis anterior tendon allograft. The Journal of Knee Surgery, 33(7), 722–727. Available from: 10.1055/s-0039-1684010 30959535

[9] Cristiani, R. , Mouton, C. , Siboni, R. , Pioger, C. & Seil, R. (2022) Failure of primary ACL repair with dynamic intraligamentary stabilization may result in a high risk of two‐stage ACL reconstruction: a case series of ten patients. Journal of Experimental Orthopaedics, 9, 79. Available from: 10.1186/S40634-022-00519-2 35976459 PMC9385901

[10] Farid, A. , Gommers, S.A. , Sierevelt, I.N. , van Eijk, F. , van Kampen, P.M. & Haverkamp, D. (2023) Graft failure and revision rate after ACL repair with dynamic intraligamentary stabilization. One‐year results of a prospective case series of 155 patients. Journal of Experimental Orthopaedics, 10(1), 52. Available from: 10.1186/s40634-023-00614-y 37145187 PMC10163193

[11] Fernández‐González, P. , Koutsou, A. , Cuesta‐Gómez, A. , Carratalá‐Tejada, M. , Miangolarra‐Page, J.C. & Molina‐Rueda, F. (2020) Reliability of Kinovea® software and agreement with a three‐dimensional motion system for gait analysis in healthy subjects. Sensors, 20, 3154. Available from: 10.3390/S20113154 32498380 PMC7308968

[12] Ferreira, A. , Saithna, A. , Carrozzo, A. , Guy, S. , Vieira, T.D. , Barth, J. et al. (2022) The minimal clinically important difference, patient acceptable symptom state, and clinical outcomes of anterior cruciate ligament repair versus reconstruction: a matched‐pair analysis from the SANTI Study Group. The American Journal of Sports Medicine, 50(13), 3522–3532. Available from: 10.1177/03635465221126171 36259683

[13] Frobell, R.B. , Roos, E.M. , Roos, H.P. , Ranstam, J. & Lohmander, L.S. (2010) A randomized trial of treatment for acute anterior cruciate ligament tears. New England Journal of Medicine, 363(4), 331–342. Available from: 10.1056/NEJMoa0907797 20660401

[14] Georgoulis, A.D. , Ristanis, S. , Moraiti, C.O. , Paschos, N. , Zampeli, F. , Xergia, S. et al. (2010) ACL injury and reconstruction: clinical related in vivo biomechanics. Orthopaedics & Traumatology, Surgery & Research: OTSR, 96(8), 119–128. Available from: 10.1016/j.otsr.2010.09.004 21036116

[15] Glasbrenner, J. , Raschke, M.J. , Kittl, C. , Herbst, E. , Peez, C. , Briese, T. et al. (2022) Comparable instrumented knee joint laxity and patient‐reported outcomes after ACL repair with dynamic intraligamentary stabilization or ACL reconstruction: 5‐year results of a randomized controlled trial. The American Journal of Sports Medicine, 50(12), 3256–3264. Available from: 10.1177/03635465221117777 36005281 PMC9527444

[16] Gokeler, A. , Benjaminse, A. , Hewett, T.E. , Lephart, S.M. , Engebretsen, L. , Ageberg, E. et al. (2012) Proprioceptive deficits after ACL injury: are they clinically relevant? British Journal of Sports Medicine, 46(3), 180–192. Available from: 10.1136/bjsm.2010.082578 21511738

[17] Gokeler, A. , Welling, W. , Benjaminse, A. , Lemmink, K. , Seil, R. & Zaffagnini, S. (2017) A critical analysis of limb symmetry indices of hop tests in athletes after anterior cruciate ligament reconstruction: a case control study. Orthopaedics & Traumatology, Surgery & Research, 103(6), 947–951. Available from: 10.1016/j.otsr.2017.02.015 28428033

[18] Grindem, H. , Snyder‐Mackler, L. , Moksnes, H. , Engebretsen, L. & Risberg, M.A. (2016) Simple decision rules can reduce reinjury risk by 84% after ACL reconstruction: the Delaware‐Oslo ACL cohort study. British Journal of Sports Medicine, 50(13), 804–808. Available from: 10.1136/bjsports-2016-096031 27162233 PMC4912389

[19] Gustavsson, A. , Neeter, C. , Thomeé, P. , Grävare Silbernagel, K. , Augustsson, J. , Thomeé, R. et al. (2006) A test battery for evaluating hop performance in patients with an ACL injury and patients who have undergone ACL reconstruction. Knee Surgery, Sports Traumatology, Arthroscopy, 14(8), 778–788. Available from: 10.1007/s00167-006-0045-6 16525796

[20] Henle, P. , Röder, C. , Perler, G. , Heitkemper, S. & Eggli, S. (2015) Dynamic Intraligamentary Stabilization (DIS) for treatment of acute anterior cruciate ligament ruptures: case series experience of the first three years. BMC Musculoskeletal Disorders, 16, 27. Available from: 10.1186/S12891-015-0484-7 25813910 PMC4341869

[21] Henle, P. , Bieri, K.S. , Brand, M. , Aghayev, E. , Bettfuehr, J. , Haeberli, J. et al. (2018) Patient and surgical characteristics that affect revision risk in dynamic intraligamentary stabilization of the anterior cruciate ligament. Knee Surgery, Sports Traumatology, Arthroscopy, 26(4), 1182–1189. Available from: 10.1007/s00167-017-4574-y 28523340

[22] Hoogeslag, R.A.G. , Brouwer, R.W. , Boer, B.C. , de Vries, A.J. & Huis in 't Veld, R. (2019) Acute anterior cruciate ligament rupture: repair or reconstruction? Two‐year results of a randomized controlled clinical trial. The American Journal of Sports Medicine, 47(3), 567–577. Available from: 10.1177/0363546519825878 30822124

[23] Hoogeslag, R.A.G. , Huis In't Veld, R. , Brouwer, R.W. , de Graaff, F. & Verdonschot, N. (2022) Acute anterior cruciate ligament rupture: repair or reconstruction? Five‐year results of a randomized controlled clinical trial. The American Journal of Sports Medicine, 50(7), 1779–1787. Available from: 10.1177/03635465221090527 35486517

[24] Kayaalp, M.E. , Sürücü, S. , Halis Çerçi, M. , Aydın, M. & Mahiroğulları, M. (2022) Anterior cruciate ligament repair using dynamic intraligamentary stabilization provides a similarly successful outcome as all‐inside anterior cruciate ligament reconstruction with a faster psychological recovery in moderately active patients. Joint Diseases and Related Surgery, 33(2), 406–413. Available from: 10.52312/jdrs.2022.631 35852201 PMC9361114

[25] Kohl, S. , Evangelopoulos, D.S. , Schär, M.O. , Bieri, K. , Müller, T. & Ahmad, S.S. (2016) Dynamic intraligamentary stabilisation: initial experience with treatment of acute ACL ruptures. The Bone & Joint Journal, 98–B(6), 793–798. Available from: 10.1302/0301-620X.98B6.35040 27235522

[26] Kotsifaki, A. , Korakakis, V. , Graham‐Smith, P. , Sideris, V. & Whiteley, R. (2021) Vertical and horizontal hop performance: contributions of the hip, knee, and ankle. Sports Health: A Multidisciplinary Approach, 13(2), 128–135. Available from: 10.1177/1941738120976363 PMC8167345 33560920

[27] Kotsifaki, A. , Van Rossom, S. , Whiteley, R. , Korakakis, V. , Bahr, R. , D'Hooghe, P. et al. (2022) Between‐limb symmetry in acl and tibiofemoral contact forces in athletes after ACL reconstruction and clearance for return to sport. Orthopaedic Journal of Sports Medicine, 10, 232596712210847. Available from: 10.1177/23259671221084742 PMC9006381 35434169

[28] Kyritsis, P. , Bahr, R. , Landreau, P. , Miladi, R. & Witvrouw, E. (2016) Likelihood of ACL graft rupture: not meeting six clinical discharge criteria before return to sport is associated with a four times greater risk of rupture. British Journal of Sports Medicine, 50(15), 946–951. Available from: 10.1136/bjsports-2015-095908 27215935

[29] Larsen, J.B. , Farup, J. , Lind, M. & Dalgas, U. (2015) Muscle strength and functional performance is markedly impaired at the recommended time point for sport return after anterior cruciate ligament reconstruction in recreational athletes. Human Movement Science, 39, 73–87. Available from: 10.1016/j.humov.2014.10.008 25461435

[30] Laughlin, W.A. , Weinhandl, J.T. , Kernozek, T.W. , Cobb, S.C. , Keenan, K.G. & O'Connor, K.M. (2011) The effects of single‐leg landing technique on ACL loading. Journal of Biomechanics, 44(10), 1845–1851. Available from: 10.1016/j.jbiomech.2011.04.010 21561623

[31] Leister, I. , Mattiassich, G. , Kindermann, H. , Ortmaier, R. , Barthofer, J. , Vasvary, I. et al. (2018) Reference values for fatigued versus non‐fatigued limb symmetry index measured by a newly designed single‐leg hop test battery in healthy subjects: a pilot study. Sport Sciences for Health, 14(1), 105–113. Available from: 10.1007/s11332-017-0410-5 29599846 PMC5866266

[32] Ma, J. , Zhang, D. , Zhao, T. , Liu, X. , Wang, J. & Zheng, H. (2020) The effects of proprioceptive training on anterior cruciate ligament reconstruction rehabilitation: a systematic review and meta‐analysis. Clinical Rehabilitation, 35(4), 506–521. 10.1177/0269215520970737 33222527

[33] Meng, J. , Xie, D. , Meng, F. , Liu, W. , Xiao, Y. , Tang, H. et al. (2023) Clinical outcomes in dynamic intraligamentary stabilization technique for anterior cruciate ligament tear: a meta‐analysis. Medicine, 102(10), e33091. Available from: 10.1097/MD.0000000000033091 36897704 PMC9997816

[34] Mir, S.M. , Hadian, M.R. , Talebian, S. & Nasseri, N. (2008) Functional assessment of knee joint position sense following anterior cruciate ligament reconstruction. British Journal of Sports Medicine, 42(4), 300–303. Available from: 10.1136/bjsm.2007.044875 18390774

[35] Miura, K. , Ishibashi, Y. , Tsuda, E. , Okamura, Y. , Otsuka, H. & Toh, S. (2004) The effect of local and general fatigue on knee proprioception. Arthroscopy: The Journal of Arthroscopic & Related Surgery, 20(4), 414–418. Available from: 10.1016/j.arthro.2004.01.007 15067282

[36] Nwachukwu, B.U. , Chang, B. , Voleti, P.B. , Berkanish, P. , Cohn, M.R. , Altchek, D.W. et al. (2017) Preoperative short form health survey score is predictive of return to play and minimal clinically important difference at a minimum 2‐year follow‐up after anterior cruciate ligament reconstruction. The American Journal of Sports Medicine, 45, 2784–2790. Available from: 10.1177/0363546517714472 28727937

[37] Nwachukwu, B.U. , Sullivan, S.W. , Rauck, R.C. , James, E.W. , Burger, J.A. , Altchek, D.W. et al. (2021) Patient‐reported outcomes and factors associated with achieving the minimal clinically important difference after ACL reconstruction: results at a mean 7.7‐year follow‐up, JBJS Open Access. 10.2106/JBJS.OA.21.00056 PMC8613365 34841188

[38] Paterno, M.V. , Huang, B. , Thomas, S. , Hewett, T.E. & Schmitt, L.C. (2017) Clinical factors that predict a second ACL injury after ACL reconstruction and return to sport: preliminary development of a clinical decision algorithm. Orthopaedic Journal of Sports Medicine, 5, 232596711774527. Available from: 10.1177/2325967117745279 PMC5753959 29318172

[39] Patras, K. , Ziogas, G. , Ristanis, S. , Tsepis, E. , Stergiou, N. & Georgoulis, A.D. (2009) High intensity running results in an impaired neuromuscular response in ACL reconstructed individuals. Knee Surgery, Sports Traumatology, Arthroscopy, 17(8), 977–984. Available from: 10.1007/s00167-009-0822-0 19495726

[40] Patras, K. , Ziogas, G. , Ristanis, S. , Tsepis, E. , Stergiou, N. & Georgoulis, A.D. (2010) ACL reconstructed patients with a BPTB graft present an impaired vastus lateralis neuromuscular response during high intensity running. Journal of Science and Medicine in Sport, 13(6), 573–577. Available from: 10.1016/j.jsams.2009.12.001 20227341

[41] Patterson, B.E. , Crossley, K.M. , Perraton, L.G. , Kumar, A.S. , King, M.G. , Heerey, J.J. et al. (2020) Limb symmetry index on a functional test battery improves between one and five years after anterior cruciate ligament reconstruction, primarily due to worsening contralateral limb function. Physical Therapy in Sport, 44, 67–74. Available from: 10.1016/j.ptsp.2020.04.031 32447259

[42] Persson, F. , Turkiewicz, A. , Bergkvist, D. , Neuman, P. & Englund, M. (2018) The risk of symptomatic knee osteoarthritis after arthroscopic meniscus repair vs partial meniscectomy vs the general population. Osteoarthritis and Cartilage, 26(2), 195–201. Available from: 10.1016/j.joca.2017.08.020 29146386

[43] Relph, N. , Herrington, L. & Tyson, S. (2014) The effects of ACL injury on knee proprioception: a meta‐analysis. Physiotherapy, 100(3), 187–195. Available from: 10.1016/j.physio.2013.11.002 24690442

[44] Ross, M.D. , Langford, B. & Whelan, P.J. (2002) Test‐retest reliability of 4 single‐leg horizontal hop tests. Journal of Strength and Conditioning Research, 16(4), 617–622. Available from: 10.1519/00124278-200211000-00021 12423195

[45] Samuelsen, B.T. , Webster, K.E. , Johnson, N.R. , Hewett, T.E. & Krych, A.J. (2017) Hamstring autograft versus patellar tendon autograft for ACL reconstruction: is there a difference in graft failure rate? A meta‐analysis of 47,613 patients. Clinical Orthopaedics & Related Research, 475(10), 2459–2468. Available from: 10.1007/s11999-017-5278-9 28205075 PMC5599382

[46] Schliemann, B. , Lenschow, S. , Domnick, C. , Herbort, M. , Häberli, J. , Schulze, M. et al. (2017) Knee joint kinematics after dynamic intraligamentary stabilization: cadaveric study on a novel anterior cruciate ligament repair technique. Knee Surgery, Sports Traumatology, Arthroscopy, 25, 1184–1190. Available from: 10.1007/s00167-015-3735-0 26239862

[47] Schliemann, B. , Glasbrenner, J. , Rosenbaum, D. , Lammers, K. , Herbort, M. , Domnick, C. et al. (2018) Changes in gait pattern and early functional results after ACL repair are comparable to those of ACL reconstruction. Knee Surgery, Sports Traumatology, Arthroscopy, 26(2), 374–380. Available from: 10.1007/s00167-017-4618-3 28674740

[48] Sorey, W. , Hagen, M.S. , Mand, S. , Sliepka, J. , Chin, K. , Schmale, G.A. et al. (2023) Effect of delayed anterior cruciate ligament reconstruction on repair of concomitant medial meniscus tears in young athletes. The American Journal of Sports Medicine, 51(2), 398–403. Available from: 10.1177/03635465221142325 36533946

[49] Strong, A. , Arumugam, A. , Tengman, E. , Röijezon, U. & Häger, C.K. (2021) Properties of knee joint position sense tests for anterior cruciate ligament injury: a systematic review and meta‐analysis. Orthopaedic Journal of Sports Medicine, 9, 232596712110078. Available from: 10.1177/23259671211007878 PMC8287371 34350298

[50] Thomeé, R. , Kaplan, Y. , Kvist, J. , Myklebust, G. , Risberg, M.A. , Theisen, D. et al. (2011) Muscle strength and hop performance criteria prior to return to sports after ACL reconstruction. Knee Surgery, Sports Traumatology, Arthroscopy, 19(11), 1798–1805. Available from: 10.1007/s00167-011-1669-8 21932078

[51] Tigerstrand Grevnerts, H. , Grävare Silbernagel, K. , Sonesson, S. , Ardern, C. , Österberg, A. , Gauffin, H. et al. (2017) Translation and testing of measurement properties of the Swedish version of the IKDC subjective knee form. Scandinavian Journal of Medicine & Science in Sports, 27(5), 554–562. Available from: 10.1111/sms.12861 28207954

[52] Weber, J. , Koch, M. , Angele, P. & Zellner, J. (2018) The role of meniscal repair for prevention of early onset of osteoarthritis. Journal of Experimental Orthopaedics, 5, 10. Available from: 10.1186/S40634-018-0122-Z 29607459 PMC5879034

[53] Wellsandt, E. , Failla, M.J. & Snyder‐Mackler, L. (2017) Limb symmetry indexes can overestimate knee function after anterior cruciate ligament injury. Journal of Orthopaedic & Sports Physical Therapy, 47(5), 334–338. Available from: 10.2519/jospt.2017.7285 28355978 PMC5483854

[54] Wren, T.A.L. , Mueske, N.M. , Brophy, C.H. , Pace, J.L. , Katzel, M.J. , Edison, B.R. et al. (2018) Hop distance symmetry does not indicate normal landing biomechanics in adolescent athletes with recent anterior cruciate ligament reconstruction. Journal of Orthopaedic & Sports Physical Therapy, 48(8), 622–629. Available from: 10.2519/jospt.2018.7817 29602303

